# Histomorphological Evaluation of the Protective Role of Vitamin D3 Against Cadmium-Induced Neurotoxicity in Albino Rats: An Experimental Study

**DOI:** 10.7759/cureus.111274

**Published:** 2026-06-22

**Authors:** Keerthy Ajay Kumar, Marimuthu P, M. Tariq Zaidi, Aijaz A Khan

**Affiliations:** 1 Anatomy, All India Institute of Medical Sciences, Raebareli, Raebareli, IND; 2 Oral and Maxillofacial Surgery, All India Institute of Medical Sciences-Central Armed Police Forces Institute of Medical Science, New Delhi, IND; 3 Anatomy, Jawaharlal Nehru Medical College, Aligarh Muslim University, Aligarh, IND; 4 Anatomy, Jawaharlal Nehru Medical College, Aligarh, IND

**Keywords:** cadmium, morphological alteration, neuroprotection, olfactory bulb, vitamin d3

## Abstract

Background

Cadmium (Cd) is one of the toxic metals found naturally in the environment and is used in industrial settings. In uncontaminated soils and freshwater, its levels are generally low. However, industrial activities, mining, phosphate fertilizers, and waste disposal can markedly increase environmental Cd concentrations, leading to chronic exposure in humans and animals. Exposure to high levels of Cd may induce harmful effects on nervous tissue. Experimental studies have demonstrated that Cd accumulates in various brain regions, including the olfactory bulb, after crossing the blood-brain barrier. This work was conducted to study the effect of Cd on the olfactory bulb of adult albino rats and the possible protective role of vitamin D3 (VD3).

Methods

A two-year experimental study was conducted in the Department of Anatomy, Jawaharlal Nehru Medical College, Aligarh Muslim University, Aligarh, after approval from the IAEC, from November 28, 2020, to November 30, 2022 (24 months). A total of 36 adult albino rats of either sex were included in the study and divided into three groups. Group I served as the control group and received normal saline. Group II served as the exposure group and received Cd chloride at a dose of 5 mg/kg/day administered subcutaneously. Group III served as the treatment group and received VD3 at a dose of 1000 IU/kg orally every two days, along with Cd chloride, for a period of four weeks. At the end of the experimental period, specimens of the olfactory bulb from all groups were collected, processed, and stained with H&E for light microscopic examination.

Results

Among the three groups, the control group showed normal architecture. The Cd chloride group showed loosening of the olfactory nerve layer, distortion of glomeruli with detached periglomerular cells occasionally interspersed with cell clusters, and vacuolar spaces in the neuropil of the external plexiform layer (EPL). Mitral cells showed morphological alterations and pericellular spaces. In the protective group, glomeruli were arranged closely, similar to those in the control group. Vacuolar spaces were negligible in the neuropil of the EPL.

Conclusions

Cd exposure causes morphological alterations in the olfactory bulb, while concurrent VD3 administration appears to reduce these changes, suggesting a neuroprotective role.

## Introduction

Cadmium (Cd) is a widely distributed environmental contaminant known for its high toxicity and persistence. Exposure to Cd through contaminated food, water, and occupational settings has been associated with a variety of diseases. As a toxic heavy metal, Cd can accumulate in biological tissues and produce harmful effects in both humans and animals [1]. In the general population, the primary nonoccupational sources of Cd exposure are dietary intake and inhalation of cigarette smoke [2]. Certain professions carry a higher risk of exposure, particularly those involving battery production, welding or soldering processes, mining, smelting, textile manufacturing, and the production of Cd-containing materials such as plastics and paints [3].

Studies have demonstrated that Cd exposure induces lipid peroxidation (LPO) in multiple tissues of adult rats, even at low to moderate doses, with especially pronounced effects observed in the brain and lungs [4]. Cd is capable of crossing into brain tissue, where it exerts neurotoxic effects, contributing to impairments in memory and disturbances in olfactory function in both human and experimental models [5,6].

The olfactory epithelium is particularly susceptible to environmental neurotoxins because of its direct exposure to the external environment. Notably, the olfactory nerve provides a pathway that can bypass the blood-brain barrier, allowing toxic substances to gain direct access to the CNS. As a result, primary olfactory neurons may serve as early targets of inhaled Cd [7]. Based on this vulnerability, the olfactory bulb was selected for histopathological evaluation in the present study. Structurally, it consists of several distinct layers arranged from superficial to deep: the olfactory nerve layer (ONL), glomerular layer (GL), external plexiform layer (EPL), mitral cell layer (MCL), internal plexiform layer (IPL), and granule cell layer (GCL).

Vitamin D3 (VD3) is a steroid hormone obtained either through dietary sources or synthesized in the skin upon exposure to ultraviolet radiation [8]. While it is well known for its role in calcium homeostasis, vitamin D also plays a significant role in the development and functioning of the CNS. Both neurons and microglial cells express vitamin D receptors, and they possess the enzymatic machinery, including 1-α-hydroxylase, necessary to convert 25(OH)D3 into its active form [9]. Furthermore, vitamin D is involved in regulating the development of neurons and oligodendrocytes, and the interaction between these cell types is essential for proper myelination during early development, as well as for remyelination following neural injury [10].

Despite existing knowledge on Cd toxicity and the neurological functions of vitamin D, there is limited information regarding the protective effects of VD3 against Cd-induced damage in the olfactory bulb. Therefore, the present study was designed to investigate both gross and histological alterations in the olfactory bulb of adult albino rats exposed to Cd chloride and to evaluate whether VD3 can provide a protective effect against such neurotoxic changes.

## Materials and methods

Study setting

The animal experimental study was carried out in the Department of Anatomy, Jawaharlal Nehru Medical College (JNMC), Aligarh Muslim University (AMU), Aligarh, India.

Ethical approval

Ethical clearance was obtained from the Institutional Animal Ethics Committee (reg. no. 401/GO/Re/S/2001/CPCSEA, dated November 28, 2020).

Animals

A total of 36 adult albino rats of the Wistar strain of either sex, weighing 200-225 g, were obtained from the Central Animal House, JNMC, AMU, Aligarh, India. The rats were housed under standard laboratory conditions in separate cages at 25°C with free access to food and water throughout the study.

Chemicals and drugs

Cd chloride (CdCl₂, 99% pure anhydrous) and VD3 were used in the study.

Experimental design

The 36 rats were randomly divided into three equal groups with an equal male-to-female ratio. Group I (control group) received sterile normal saline subcutaneously. Group II (exposure group) received Cd chloride dissolved in 0.9% saline at a dose of 5 mg/kg body weight subcutaneously daily for four weeks [11]. Group III (treatment group) received Cd chloride at the same dose and duration, along with VD3 at 1000 IU/kg orally every two days [12].

At the end of the experimental period, the rats were euthanized with a high dose of diethyl ether and perfusion-fixed with 10% formalin. The brains were dissected to examine gross changes, and the olfactory bulbs were removed and placed in 10% formalin for 48 hours for further fixation. The olfactory bulbs were then processed for paraffin embedding. Tissue blocks were prepared, and 10-µm-thick sections were obtained and stained with H&E for histopathological examination under light microscopy.

## Results

Gross observations

Brains from the control group displayed normal coloration (Figure 1a). In contrast, Cd chloride exposure led to a purplish hue and noticeable shrinkage of brain tissue (Figure 1b). In the group treated with VD3 alongside Cd chloride, brain color was largely preserved and resembled that of the control group (Figure 1c), suggesting a protective effect at the gross level.

**Figure 1:**
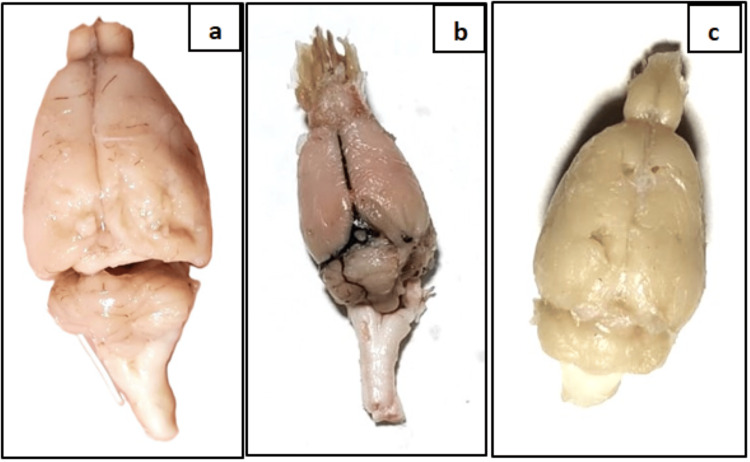
(a) Brain specimen from the control group showing normal color. (b) Brain specimen from the Cd exposure group showing a purplish hue and shrinkage. (c) Brain specimen from the treatment group showing largely preserved color similar to the control group. Cd, cadmium

Histological observations

Control Group

Histological sections of the olfactory bulb revealed the typical layered arrangement from the surface inward: ONL, GL, EPL, MCL, IPL, and GCL (Figure 2). The ONL contained parallel unmyelinated axons. Glomeruli in the GL were closely packed and surrounded by periglomerular cells (Figure 3). The EPL consisted predominantly of neuropil with few scattered cells. The MCL formed a thin band of large pyramidal mitral cells. The IPL contained small- to medium-sized cells, and the GCL exhibited evenly distributed granule neurons, some arranged in clusters.

**Figure 2:**
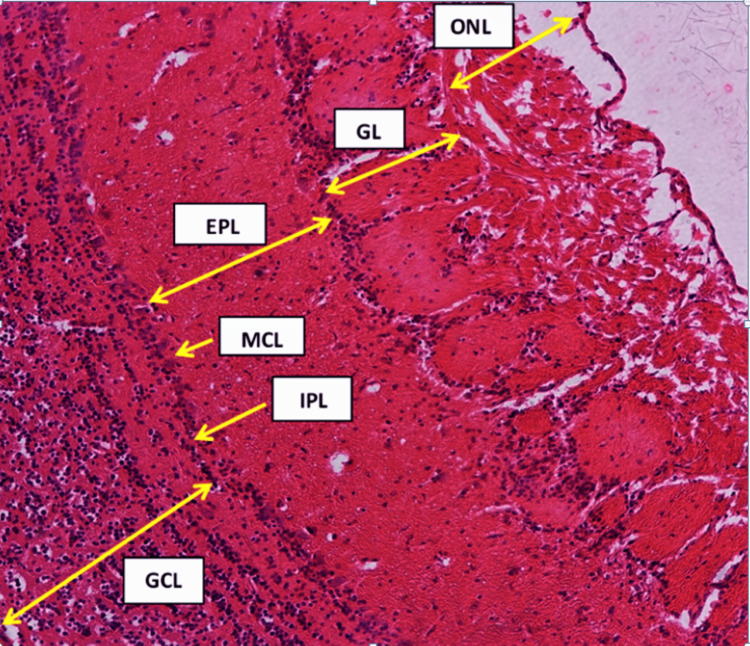
Photomicrograph of the olfactory bulb from the control group showing six laminae: ONL, GL, EPL, MCL, IPL, and GCL. H&E ×100. EPL, external plexiform layer; GCL, granule cell layer; GL, glomerular layer; IPL, internal plexiform layer; MCL, mitral cell layer; ONL, olfactory nerve layer

**Figure 3:**
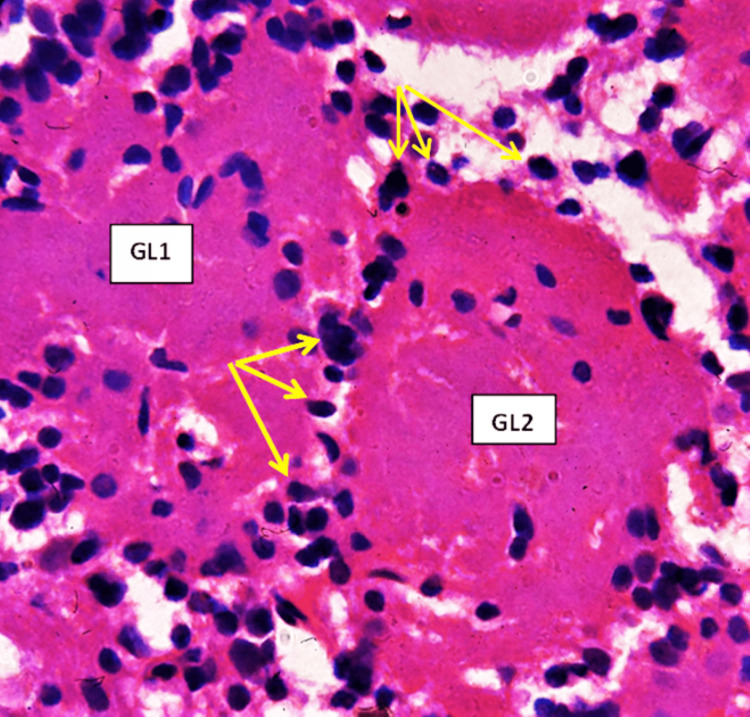
Photomicrograph of the olfactory bulb from the control group showing two glomeruli (GL1 and GL2) with uniformly distributed periglomerular cells (→) in the surrounding region. H&E ×1000. GL, glomerular layer

Cd Chloride Exposure Group

Cd exposure disrupted the normal architecture of the olfactory bulb. The ONL displayed loosening of axonal fibers. Glomeruli were irregular in shape with detached periglomerular cells, and interglomerular spaces were expanded (Figure 4). The EPL showed numerous vacuoles within the neuropil. Cells within the external plexiform, mitral, and GCLs exhibited a disorganized arrangement and pericellular spaces (Figure 5).

**Figure 4:**
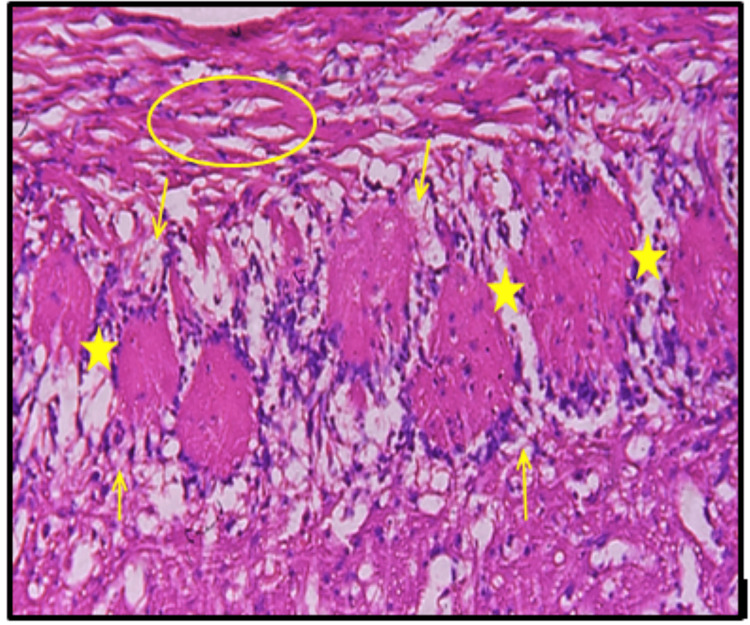
Photomicrograph of the olfactory bulb from the Cd exposure group showing loosening of nerve fibers (○) in the ONL, increased interglomerular space (*), and a reticular appearance of nerve fibers (→) adjacent to the glomeruli involving superficial and deeper regions. H&E ×200. Cd, cadmium; ONL, olfactory nerve layer

**Figure 5:**
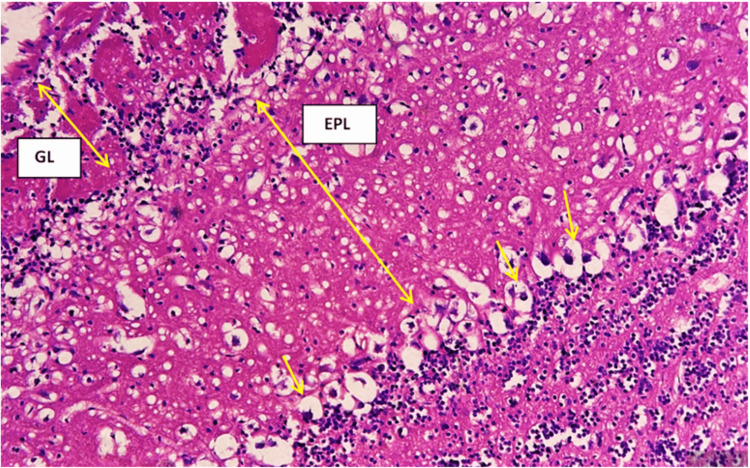
Photomicrograph of the olfactory bulb from the Cd exposure group showing the GL with detached periglomerular cells and the EPL with variably sized vacuolar spaces in the neuropil. Pericellular spaces (→) are also present around mitral cells. H&E ×100. Cd, cadmium; EPL, external plexiform layer; GL, glomerular layer

Treatment Group Cd + VD3

In this group, the ONL was largely intact. Most glomeruli were closely positioned and surrounded by periglomerular cells. Vacuolation in the EPL was minimal. Cellular morphology and arrangement in the external plexiform, mitral, and GCLs were preserved, with fewer pericellular spaces compared to the Cd-only group (Figure 6).

**Figure 6:**
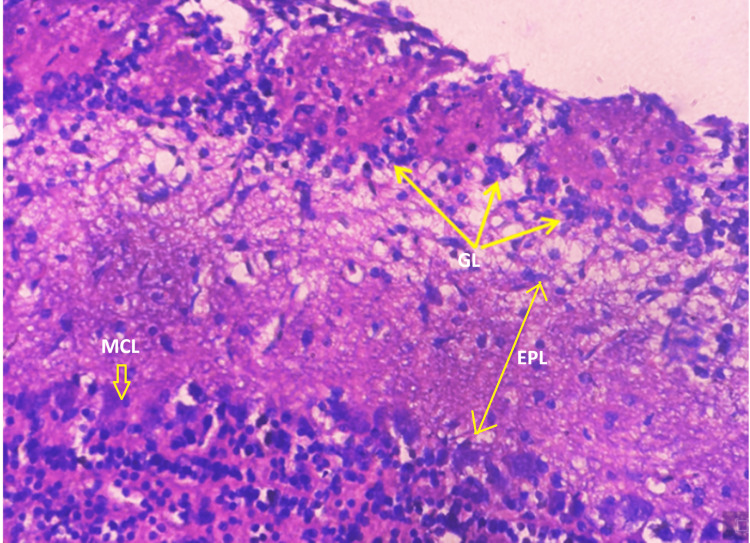
Photomicrograph of the olfactory bulb from the treatment group showing closely arranged glomeruli with surrounding periglomerular cells in the GL. The EPL shows minimal vacuolar spaces in the neuropil, and the MCL shows minimal pericellular spaces around mitral cells. H&E ×200. EPL, external plexiform layer; GL, glomerular layer; MCL, mitral cell layer

Figure 7 depicts the comparison between the control, Cd exposure, and treatment groups. Figure 7A shows the normal architecture of the olfactory bulb in the control group. Figure 7B shows detached periglomerular cells in the GL, vacuolar spaces in the neuropil of the EPL, and pericellular spaces around mitral cells in the olfactory bulb of the Cd exposure group. Figure 7C shows closely arranged glomeruli with surrounding periglomerular cells, minimal vacuolar spaces in the EPL, and minimal pericellular spaces around mitral cells in the olfactory bulb of the treatment group.

**Figure 7:**
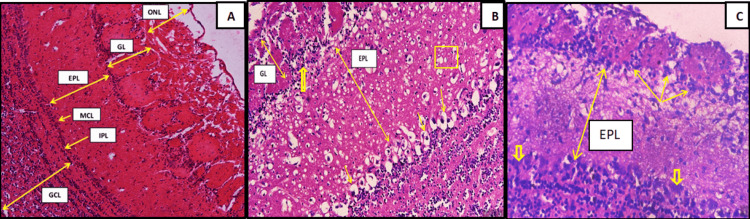
(A) Photomicrograph of the OB from the control group showing normal architecture in all six layers (ONL, GL, EPL, MCL, IPL, and GCL). (B) Photomicrograph of the OB from the Cd exposure group showing detached periglomerular cells (⇑) in the GL, increased vacuolar spaces in the neuropil (▭) of the EPL, and pericellular spaces (→) around mitral cells. (C) Photomicrograph of the OB from the treatment group showing closely arranged glomeruli with surrounding periglomerular cells (→), minimal vacuolar spaces in the neuropil of the EPL, and minimal pericellular spaces around mitral cells (⇓). Cd, cadmium; EPL, external plexiform layer; GCL, granule cell layer; GL, glomerular layer; IPL, internal plexiform layer; MCL, mitral cell layer; OB, olfactory bulb; ONL, olfactory nerve layer

## Discussion

The findings of the present study indicate that administration of Cd chloride resulted in marked structural alterations in the olfactory bulb of adult albino rats, particularly disruption of its normal laminar arrangement. Following exposure, the glomeruli exhibited morphological distortion characterized by widening of interglomerular spaces, along with clusters of periglomerular cells displaced from their typical positions. Comparable observations have been reported by Patel et al., who documented significant histopathological changes in the olfactory bulb of Cd-exposed zebrafish, including expanded periglomerular spaces and areas of increased vascular congestion [13].

Additionally, the current study revealed the presence of variably sized vacuolated areas distributed throughout the neuropil of the EPL, accompanied by prominent pericellular spaces. Similar changes were observed in the MCL and GCL, where both the large pyramidal mitral neurons and smaller rounded granule cells displayed noticeable pericellular clearing.

Our study further demonstrated that Cd exposure led to disorganization and loosening of nerve fibers, along with a reduction in glomerular structures within the olfactory bulb, which appeared to be replaced by aggregates of periglomerular cells. Supporting these findings, Czarnecki et al. reported that Cd exposure can impair axonal projections extending from the olfactory epithelium to the olfactory bulb [14].

Vitamin D exists in two principal forms. Vitamin D2 is produced when the plant sterol ergosterol, found in sun-exposed mushrooms, undergoes ultraviolet irradiation, whereas VD3 is synthesized in human skin upon exposure to ultraviolet B radiation and is considered the physiologically predominant form [15]. Al Rahoo and Dabdoub investigated the protective effects of VD3 against Cd chloride-induced liver toxicity in albino rats. Their histological analysis of liver tissue from CdCl₂-treated animals revealed pronounced degenerative alterations, including dilation of the central vein, hemorrhagic changes, necrosis of hepatocytes arranged in irregular cords, and sinusoidal dilatation with internal bleeding [12]. These pathological changes are likely related to the generation of reactive oxygen species, which promote oxidative damage through enhanced LPO [16]. In contrast, liver sections from rats treated with VD3 showed marked histological improvement, approaching the normal architecture observed in control animals [12].

In the present study, VD3 demonstrated a clear protective effect against Cd-induced histopathological damage in the olfactory bulb. Coadministration of VD3 with Cd chloride led to noticeable restoration of olfactory bulb architecture, with only minimal disruption of its layered organization. The ONL remained largely preserved, and the glomeruli retained near-normal structure with appropriately positioned periglomerular cells and minimal interglomerular spacing, comparable to controls. Furthermore, vacuolation within the EPL was significantly reduced, and pericellular spaces in both the mitral and GCLs were less prominent. These observations strongly suggest that VD3 mitigates Cd-induced neurotoxicity in the olfactory bulb of adult albino rats. This finding is clinically significant and may support future studies evaluating the potential use of VD3 in individuals exposed to Cd in industrial and occupational settings.

The neuroprotective effects of vitamin D are attributed to its ability to regulate neurotrophic factors and reduce oxidative stress. Neurotrophic factors play a vital role in the growth, differentiation, and survival of neuronal and glial cells. Vitamin D has been shown to enhance the expression of several key neurotrophic molecules, including nerve growth factor, brain-derived neurotrophic factor, glial cell line-derived neurotrophic factor, and neurotrophin-3 [17].

There are certain limitations in our study. Although histopathological changes were evaluated, oxidative stress markers such as LPO, antioxidant enzyme activity, and apoptotic markers were not assessed biochemically. Therefore, the exact molecular mechanisms underlying Cd toxicity and VD3 protection could not be confirmed.

## Conclusions

Cd chloride causes significant structural damage to the olfactory bulb, including loss of laminar organization, glomerular degeneration, and increased pericellular and vacuolar spaces. VD3 supplementation demonstrates a strong protective effect by preserving olfactory bulb architecture, maintaining glomerular and periglomerular integrity, and minimizing Cd-induced histopathological changes. These findings support the therapeutic potential of VD3 in preventing heavy metal-induced toxicity in the olfactory bulb.
